# Robotic Versus Conventional Primary Total Knee Arthroplasty: Achievement of Minimal Clinically Important Difference at One-Year Follow-Up

**DOI:** 10.7759/cureus.106726

**Published:** 2026-04-09

**Authors:** Adarsh Venugopal, Jai Thilak

**Affiliations:** 1 Orthopedic Surgery, Amrita Hospital, Kochi, IND; 2 Orthopedics, Amrita Institute of Medical Sciences and Research Center, Kochi, IND

**Keywords:** knee arthroplasty, koos-jr, mcid, proms, robotic tka

## Abstract

Background: Total knee arthroplasty (TKA) is an effective treatment for end-stage knee osteoarthritis. However, conventional jig-based TKA (C-TKA) techniques often lack reproducibility in achieving balanced flexion-extension gaps and consistent mediolateral ligament tension. Robotic-assisted TKA (R-TKA) has emerged as a potential solution to improve accuracy in prosthesis positioning, gap balancing, and soft tissue management. This study compared R-TKA and C-TKA using validated patient-reported outcome measures (PROMs), including KOOS JR (Knee Injury and Osteoarthritis Outcome Score for Joint Replacement), PROMIS (Patient-Reported Outcomes Measurement Information System) Physical Health (PH), and PROMIS Mental Health (MH), to evaluate achievement of the minimal clinically important difference (MCID).

Methods: A retrospective cohort study was conducted on patients who underwent primary TKA. Preoperative and one-year postoperative scores for KOOS JR, PROMIS PH, and PROMIS MH were analyzed. MCID achievement was defined using the distribution-based method.

Results: Both R-TKA and C-TKA groups demonstrated significant postoperative improvements across all PROMs. At the one-year follow-up, 100% of patients in both cohorts achieved the MCID for the KOOS JR score. Similarly, high rates of MCID achievement were observed for PROMIS PH and PROMIS MH in both groups. While the R-TKA group showed a higher percentage improvement in KOOS JR scores compared to the C-TKA group, the differences between the two techniques were not statistically significant.

Conclusion: Both R-TKA and C-TKA result in excellent clinical outcomes and high rates of MCID achievement at one year. While R-TKA showed a trend toward greater functional improvement in joint-specific measures, it did not demonstrate statistical superiority over conventional methods in this cohort.

## Introduction

Total knee arthroplasty (TKA) is a highly effective treatment for symptomatic end-stage knee osteoarthritis. The number of TKA procedures performed has significantly increased in recent years, with projections for continued growth [1]. Despite advancements in surgical techniques and implant designs, approximately 10% of patients remain dissatisfied following TKA [2,3]. Optimal TKA outcomes, characterized by prosthesis function, stability, and longevity, are critically dependent on accurate component placement, precise gap balancing, appropriate ligament tensioning, and protection of the surrounding articular soft tissue envelope [4,5].

Currently, TKA can be performed using either conventional jig-based techniques or robotic-assisted approaches. Conventional jig-based TKA (C-TKA) relies heavily on surgeon skill and experience, and key steps such as gap balancing and mediolateral ligament tensioning often exhibit poor reproducibility. Furthermore, C-TKAs utilize manually controlled saw blades and handheld instruments, which, despite efforts to safeguard periarticular soft tissues, can inadvertently lead to damage to supporting ligaments. Such soft tissue injury may impede postoperative healing and function, reduce implant stability, and potentially shorten implant lifespan [6].

Robotic-assisted TKA (R-TKA) was developed to mitigate these limitations by enhancing precision in implant positioning, gap balancing, and ligament tensioning. This technology leverages preoperative imaging and 3D reconstruction of the patient's native knee anatomy to calculate a haptic "safe window" for optimal bone resection, implant sizing, and positioning [7-9]. The haptic boundary limits saw blade action, thereby minimizing the risk of soft tissue injury [10]. Previous studies indicate that R-TKA is associated with more accurate implant positioning compared to C-TKA [10-12]. However, there is a paucity of studies directly comparing functional outcomes between robotically assisted (often kinematically aligned) TKA and conventional jig-based (mechanically aligned) TKA.

To address this gap in the literature, this retrospective cohort study aimed to determine differences in functional outcomes between R-TKA and C-TKA patients over a one-year follow-up period. Our hypothesis was that patients undergoing R-TKA would achieve superior functional outcomes compared to those undergoing conventional mechanically-aligned TKA.

Patient-reported outcome measures (PROMs) are indispensable tools for evaluating health conditions and assessing treatment responses from the patient's perspective. PROMs are particularly useful for gauging the effectiveness of TKA for symptomatic knee osteoarthritis and for comparing functional outcomes between R-TKA and C-TKA. While an improvement in PROMs after a procedure indicates a patient's response, a truly clinically important difference signifies a change that patients would perceive as worthwhile, leading them to consider repeating the intervention if given the choice [13]. The minimal clinically important difference (MCID) represents the smallest change in a PROM score that is meaningful to a patient. Patients whose PROMs improve by a value equal to or greater than the MCID are often referred to as "responders," indicating they have experienced a significant and perceptible benefit from the intervention.

## Materials and methods

**Figure 1:**
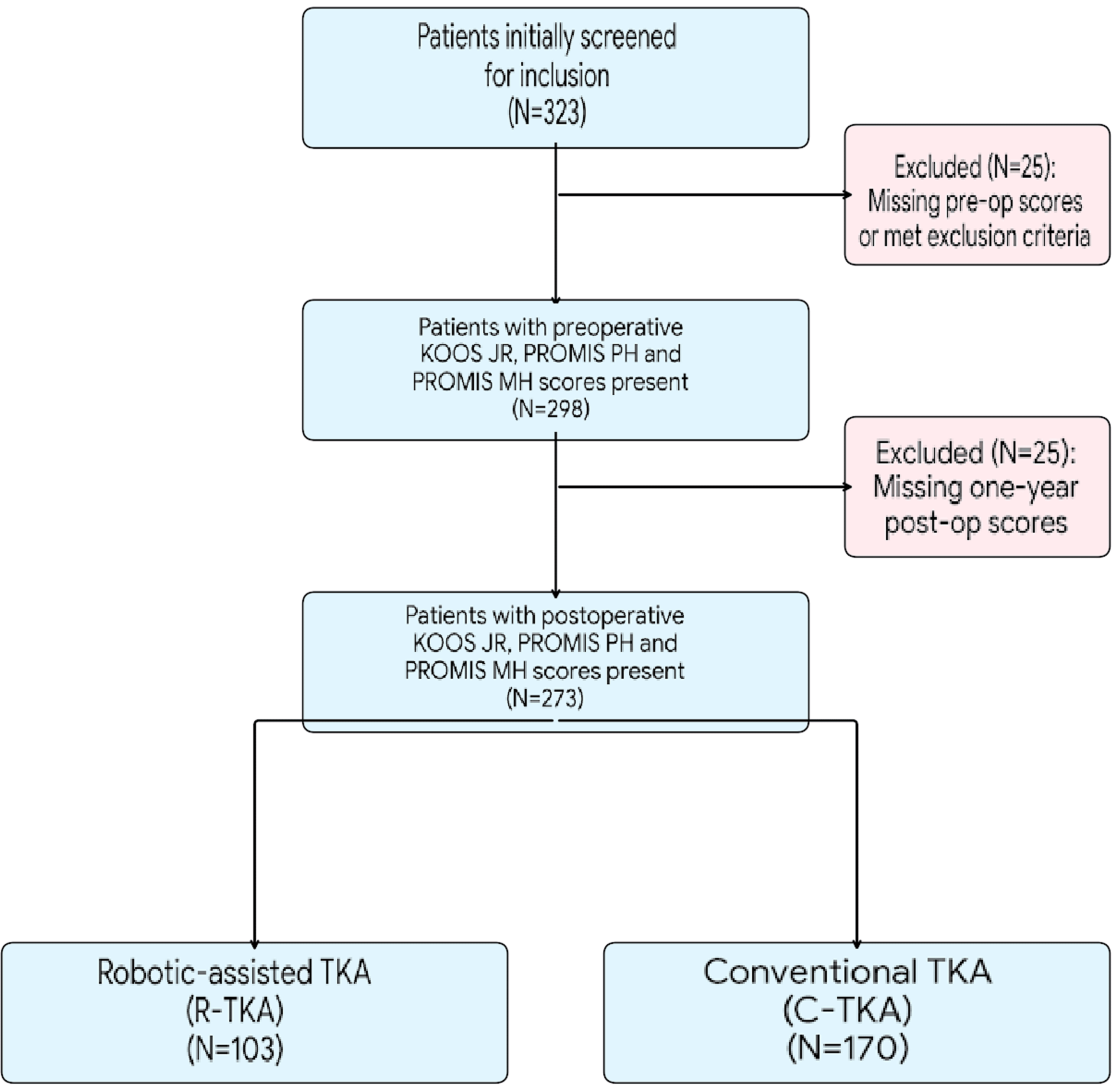
Screening criteria for patient selection This flowchart illustrates the retrospective screening and inclusion process for the study cohort. Starting from an initial pool of 323 patients, the diagram details the sequential exclusion of 50 patients due to missing preoperative or one-year postoperative data (KOOS JR, PROMIS PH, or PROMIS MH scores). The final study population of 273 patients is categorized into two cohorts: 103 patients who underwent robotic-assisted total knee arthroplasty (R-TKA) and 170 patients who underwent conventional jig-based total knee arthroplasty (C-TKA). KOOS JR: Knee Injury and Osteoarthritis Outcome Score for Joint Replacement; PROMIS PH: Patient-Reported Outcomes Measurement Information System Physical Health; PROMIS MH: Patient-Reported Outcomes Measurement Information System Mental Health

The study utilized a retrospective cohort design to evaluate patients who underwent primary TKA at our institution between March 2020 and October 2022. The screening process was conducted systematically, beginning with an initial identification of 323 potential participants. Following the application of strict inclusion and exclusion criteria, such as the removal of cases involving revision surgery, traumatic arthroplasty, or incomplete follow-up data, a final cohort of 273 patients was established. These patients were then categorized into two distinct groups based on the surgical technique employed: 103 patients received R-TKA using an image-free system, while 170 patients underwent C-TKA (Figure 1).

All necessary data were obtained from the institutional database. This study received approval from the institutional review board.

Surgical techniques and implants

R-TKAs were performed using a MAKO robotic-assisted surgical system (Stryker, Kalamazoo, MI, USA). For all R-TKAs, the surgeon utilized a cemented cruciate-retaining (CR) implant (Triathlon, Stryker) following an adjusted mechanical alignment sequence. In contrast, the conventional group received a combination of CR and posterior-stabilizing (PS) implants, with the choice of prosthesis often dictated by patient insurance coverage and financial considerations. All procedures across both cohorts were performed by a single, high-volume fellowship-trained orthopedic surgeon to minimize interoperator variability. Postoperative protocols, including pain management and physical rehabilitation, were standardized across both groups to ensure that the primary variable remained the surgical technique itself.

Data collection and outcome measures

Demographic data were standardized and collected from the hospital information system. The primary outcomes were preoperative and one-year postoperative scores for the Knee Injury and Osteoarthritis Outcome Score for Joint Replacement (KOOS JR), Patient-Reported Outcomes Measurement Information System (PROMIS) Physical Health (PH), and PROMIS Mental Health (MH). Patient-reported functional outcomes were assessed using three validated instruments: the KOOS JR, the PROMIS PH, and the PROMIS MH. The KOOS JR is an open-access, shortened version of the original KOOS score, developed by the Centers for Medicare and Medicaid Services (CMS) [14]. The PROMIS PH and PROMIS MH scales are part of the PROMIS Global Health v1.2 battery, which is an open-access resource developed and validated by the National Institutes of Health (NIH) [15]. As these tools are designated for open-access research and clinical use, formal permission for their utilization was not required.

The MCID for each PROM was determined using the distribution method, specifically by taking one-half of the standard deviation (SD) of the preoperative PROM scores. An increase in postoperative scores equal to or greater than the calculated MCID was considered to represent MCID achievement.

Exclusion criteria

Patients were excluded if they underwent partial joint arthroplasty, arthroplasty due to a traumatic event, revision arthroplasty, postoperative complications, increased operating time (as a proxy for surgical complexity or unforeseen issues), multiple joint replacements within the selected study period, or missing or incomplete data.

Data analysis

We included all available patients who met the predefined inclusion and exclusion criteria and had the required data for this retrospective study. Statistical analyses were performed using IBM SPSS version 20.00 (IBM Corp., Armonk, NY, USA).

Continuous variables were described using mean ± SD, median, and interquartile range (IQR). These variables were compared between groups using either an independent samples t-test or the Mann-Whitney U test, depending on the assessment of normality assumptions.

Categorical variables are presented using counts and percentages. Comparisons between groups for categorical data were made using the chi-squared test or Fisher's exact test, based on expected cell counts. A p-value of less than 0.05 (p < 0.05) was considered statistically significant.

## Results

Patient demographics and baseline characteristics

A total of 273 patients were included in the final analysis, with 103 patients in the R-TKA group and 170 patients in the C-TKA group. The screening process and cohort allocation are detailed in Figure 1. The mean age of the R-TKA group was 65.5 ± 7.2 years, while that of the C-TKA group was 66.8 ± 8.1 years. There were no statistically significant differences between the two groups regarding age, gender distribution, or body mass index (BMI). Baseline preoperative functional scores (KOOS JR, PROMIS PH, and PROMIS MH) were also comparable between groups, as shown in Table 1.

**Table 1:** Univariate analysis of preoperative demographics, comparing characteristics between the R-TKA and C-TKA groups This table presents the univariate analysis of preoperative demographic data for the C-TKA and R-TKA cohorts. Categorical data (sex) are presented as frequencies and percentages, with the comparison performed using the chi-squared test. Continuous data (age) are presented as mean ± standard deviation (SD), median, and range; the comparison of means was performed using the independent samples t-test (reported as t-statistic). A p-value < 0.05 was considered statistically significant. A single hyphen ("-") indicates that a separate test statistic is not applicable for that specific sub-row. TKA: total knee arthroplasty; C-TKA: conventional jig-based total knee arthroplasty; R-TKA: robotic-assisted total knee arthroplasty

Parameter	C-TKA (n = 170)	R-TKA (n = 103)	Statistical analysis (test statistic, p-value)
Sex (female)	130 (76.5%)	81 (78.6%)	Chi square = 0.17, p = 0.678
Sex (male)	40 (23.5%)	22 (21.4%)	-
Age (years)	-	-	-
Mean ± SD	64.39 ± 7.58	65.20 ± 7.51	t = 0.86, p = 0.392
Median	64	65	-
Range (Min-Max)	51-84	52-84	-

Functional outcome measures

Both surgical cohorts demonstrated significant improvements in all PROMs at the one-year follow-up. In the R-TKA group, the mean KOOS JR score improved from a preoperative value of 15.29 to a postoperative value of 58.12. In the C-TKA group, the mean score improved from 18.31 to 55.67. While the R-TKA group showed a higher percentage of improvement in KOOS JR scores (280% vs. 204% in C-TKA), the difference between the groups did not reach statistical significance (p > 0.05). Similar trends of improvement were observed for the PROMIS PH and PROMIS MH scores for both groups. These comparative mean scores and their associated p-values are summarized in Table 2.

**Table 2:** Univariate comparison of PROMs between robotic and nonrobotic total knee arthroplasty This table summarizes the functional recovery outcomes at the one-year primary endpoint for both surgical groups. It displays the mean postoperative scores and the calculated percentage of improvement from baseline for the KOOS JR, PROMIS PH, and PROMIS MH scales. Although the R-TKA group exhibited a higher mean percentage improvement in joint-specific function (280% vs. 204%), p-values from independent t-tests are included to show that these differences did not reach statistical significance (p > 0.05), suggesting comparable efficacy between robotic and conventional methods in this study population. p-value < 0.05 was considered to be statistically significant. C-TKA: conventional jig-based total knee arthroplasty; R-TKA: robotic-assisted total knee arthroplasty; KOOS JR: Knee Injury and Osteoarthritis Outcome Score for Joint Replacement; PROMIS PH: Patient-Reported Outcomes Measurement Information System Physical Health; PROMIS MH: Patient-Reported Outcomes Measurement Information System Mental Health; PROMs: patient-reported outcome measures

Covariate	Statistics	Robotic (R-TKA) (n = 103)	Conventional (C-TKA) (n = 170)	Statistical analysis (test statistic, p-value)
Pre-op KOOS JR	Mean ± SD	15.29 ± 16.65	18.31 ± 16.60	t = -1.46, p = 0.174
	Median (Q1, Q3)	8.29 (0, 31.3)	15.93	-
Pre-op PROMIS MH	Mean ± SD	68.16 ± 5.55	68.34 ± 5.52	t = -0.26, p = 0.792
	Median (Q1, Q3)	67.7	69	-
Pre-op PROMIS PH	Mean ± SD	30.90 ± 3.62	31.52 ± 3.29	t = -1.45, p = 0.153
	Median (Q1, Q3)	31.5 (28.7, 32.9)	32.1	-
Post-op KOOS JR	Mean ± SD	77.06 ± 11.78	73.03 ± 11.76	t = 2.74, p = 0.007
	Median	76.33	70.7	-
Post-op PROMIS MH	Mean ± SD	49.39 ± 5.45	50.56 ± 5.32	t = -1.75, p = 0.083
	Median	50.3	52	-
Post-op PROMIS PH	Mean ± SD	43.94 ± 4.39	43.58 ± 4.23	t = 0.67, p = 0.500
	Median	43.3	43.3	-

Achievement of MCID

**Figure 2:**
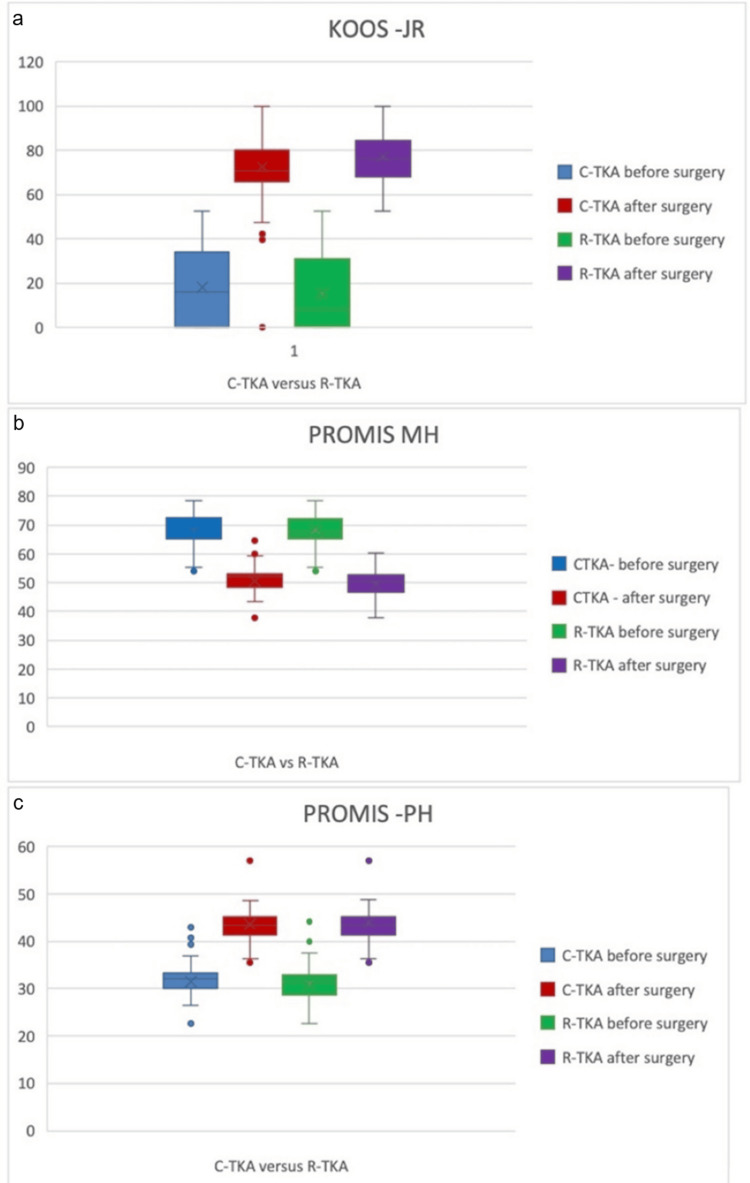
Comparison between preoperative and postoperative scores: (a) KOOS JR, (b) PROMIS MH, and (c) PROMIS PH scores in R-TKA and C-TKA This multi-panel bar chart presents the functional recovery data for patients undergoing R-TKA (N = 103) and C-TKA (N = 170). The figure displays a side-by-side comparison of mean preoperative and one-year postoperative scores across three validated metrics: the joint-specific KOOS JR (a) and the broader PROMIS PH and PROMIS MH scales (b, c). Both surgical cohorts demonstrated statistically significant improvements (p < 0.05) from baseline to the one-year follow-up in all categories. Notably, while the R-TKA group exhibited a numerically higher percentage of improvement in KOOS JR scores (280% vs. 204% in the C-TKA group), the intergroup analysis of postoperative means showed no statistically significant difference between the two techniques. This visual representation underscores that both R-TKA and C-TKA are highly effective at restoring function and improving quality of life, with comparable clinical outcomes at the primary one-year endpoint. C-TKA: conventional jig-based total knee arthroplasty; R-TKA: robotic-assisted total knee arthroplasty; KOOS JR: Knee Injury and Osteoarthritis Outcome Score for Joint Replacement; PROMIS PH: Patient-Reported Outcomes Measurement Information System Physical Health; PROMIS MH: Patient-Reported Outcomes Measurement Information System Mental Health

The primary endpoint of the study was the achievement of the MCID at one year. For the KOOS JR score, 100% of patients in both the R-TKA and C-TKA groups achieved the MCID threshold. Regarding PROMIS PH, 98.1% of the R-TKA group and 97.6% of the C-TKA group reached the MCID. For PROMIS MH, the achievement rates were 99.0% and 98.8%, respectively. No statistically significant difference was found between the two techniques in terms of the proportion of patients achieving MCID across any of the three metrics. The distribution of MCID achievement is illustrated in Figures 2a-2c.

Table 3 details the comparison of MCID achievement between the R-TKA and C-TKA groups. The MCID was calculated as half the SD of the preoperative scores for each PROM.

**Table 3:** Univariate analysis of MCID achievement in robotic versus conventional TKA This table summarizes the achievement rates of the minimal clinically important difference (MCID) for the KOOS JR, PROMIS PH, and PROMIS MH scores at the one-year follow-up. Data are presented as frequencies and percentages. Statistical comparisons between the R-TKA and C-TKA cohorts were performed using the chi-squared test, with Fisher’s exact test applied where cell counts were low. For KOOS JR, a statistical test was not applicable as both groups reached 100% achievement. A p-value < 0.05 was considered statistically significant. A single hyphen ("-") indicates where a test statistic is not applicable. TKA: total knee arthroplasty; C-TKA: conventional jig-based total knee arthroplasty; R-TKA: robotic-assisted total knee arthroplasty; KOOS JR: Knee Injury and Osteoarthritis Outcome Score for Joint Replacement; PROMIS PH: Patient-Reported Outcomes Measurement Information System Physical Health; PROMIS MH: Patient-Reported Outcomes Measurement Information System Mental Health

MCID measure	Achievement	R-TKA (n = 103)	C-TKA (n = 170)	Statistical analysis (test statistic, p-value)
KOOS JR MCID	Yes	103 (100%)	170 (100%)	-
	No	0 (0%)	0 (0%)	-
PROMIS PH MCID	Yes	100 (97.1%)	166 (97.6%)	Chi square = 0.08, p = 1.00
	No	3 (2.9%)	4 (2.4%)	-
PROMIS MH MCID	Yes	102 (99.0%)	169 (99.4%)	Chi square = 0.14, p = 1.00
	No	1 (1.0%)	1 (0.6%)	-

The analysis of patient demographics revealed that the R-TKA and C-TKA groups were well-matched at baseline. The mean age of the robotic group was 65.20 ± 7.51 years, which did not differ significantly from the mean age of 64.39 ± 7.58 years observed in the conventional group (t = 0.86, p = 0.392). Similarly, the distribution of female patients was comparable between the R-TKA (78.6%) and C-TKA (76.5%) cohorts (chi square = 0.17, p = 0.678). Preoperative functional status, assessed through the KOOS JR and PROMIS scores, also showed no statistically significant differences, providing a consistent baseline for evaluating postoperative recovery.

At the one-year primary endpoint, both surgical techniques resulted in substantial improvements across all validated PROMs. The mean KOOS JR score in the R-TKA group improved significantly from a preoperative baseline of 15.29 to a postoperative score of 77.06. Although the R-TKA group achieved a numerically higher mean postoperative KOOS JR score compared to the 73.03 observed in the C-TKA group, the difference was found to be statistically significant (t = 2.74, p = 0.007). However, when evaluating the broader measures of physical and mental health through the PROMIS PH and PROMIS MH scales, both groups exhibited nearly identical recovery patterns with no statistically significant variance between the robotic and conventional approaches.

## Discussion

From a surgical perspective, R-TKA offers several perceived advantages over conventional methods, including enhanced preoperative planning, reduced operative errors, increased precision in implant placement, and improved operating theater ergonomics [8,9,16-22]. These technical benefits are theorized to contribute to greater cost efficiency over time, particularly as the integration of artificial intelligence and machine learning suggests significant future potential for the platform. Since the introduction of the current generation of robotic systems in 2017, there has been considerable academic interest in whether these technologies demonstrably offer superior benefits over C-TKA regarding postoperative pain, functional outcomes, and procedural costs.

Our study contributes to this ongoing discourse by specifically comparing the achievement of the MCID between patients undergoing R-TKA and C-TKA. The demographic characteristics of our cohort, which had a mean age of 69.7 years and a notable female predominance (77% overall), align closely with existing literature. For instance, Kayani et al. reported a similar mean age of 69.7 years with a high proportion of female patients [6]. While some studies, such as those by Kim et al. and Khlopas et al., reported slightly younger mean ages, the consistent female predominance across these studies mirrors the population trends observed in our institutional data [23-25].

A striking finding in our cohort was the mean preoperative KOOS JR scores of 15.29 for R-TKA and 18.31 for C-TKA. These figures are considerably lower than those reported by Shaw et al. and Khan et al., whose cohorts presented with preoperative scores in the mid-40s [19,25]. The remarkably low baseline scores in our study are likely attributable to the stringent screening criteria for total knee replacement at our institution, which selects for a patient population with severe functional limitations and end-stage disease prior to surgical intervention. Despite these low baseline values, both groups demonstrated excellent clinical outcomes at the one-year follow-up. Every patient in both the R-TKA and C-TKA groups achieved the MCID for KOOS JR, while achievement rates for PROMIS MH and PROMIS PH exceeded 97% across both cohorts. This high rate of success likely reflects the substantial functional gains experienced by patients starting from a state of severe preoperative limitation.

While our study found similar MCID achievement rates at one year, earlier research has suggested potential short-term advantages for robotic assistance. Kayani et al. and Marchand et al. both observed significantly better pain and satisfaction scores in the early postoperative period for R-TKA patients [6,26]. However, our findings align more closely with Mitchell et al., who noted that while R-TKA may offer early benefits-such as lower opioid requirements and shorter hospital stays-these differences often dissipate by the one-year mark [27]. Similarly, Samuel et al. and Liow et al. found that despite differences in operative time or early recovery metrics, one-year PROMs were largely equivalent between the two techniques [28,29].

Analyzing PROMs through the lens of MCID provides a more patient-centric evaluation of success than raw score comparisons alone. Although our data showed similar MCID achievement trends, a divergence was noted in the magnitude of improvement. R-TKA patients exhibited a 279% increase in KOOS JR scores compared to 204% in the C-TKA group. The disparity between these joint-specific improvements and the more modest 4% difference observed in PROMIS PH scores suggests that the specificity of the assessment tool significantly influences the results. This highlights a critical need for the development of more sensitive and specialized tools to detect subtle yet potentially meaningful differences in surgical outcomes.

Strengths and limitations

The strengths of this study include the involvement of a single expert surgeon experienced in both R-TKA and C-TKA, which effectively minimizes intersurgeon variability. Furthermore, the use of a defined patient cohort from a consistent geographic region and the application of standardized preoperative and postoperative rehabilitation protocols helped to reduce potential confounding variables.

However, several limitations must be acknowledged. The R-TKA group received only CR implants, whereas the C-TKA group received a mix of CR and PS designs based on insurance policies and patient affordability. While current meta-analyses suggest no significant functional differences between these designs in primary TKA, this variation may introduce a degree of socioeconomic selection bias. Additionally, our analysis was limited to a single postoperative timepoint at one year, which precludes the evaluation of short-term recovery trends or long-term durability. The study also did not account for operative time or specific postoperative complications. Finally, the relatively novel nature of robotic technology at our institution resulted in a smaller sample size for the R-TKA group, and the potential influence of unmeasured confounders, such as socioeconomic status, remains a factor for consideration in future research.

## Conclusions

In this retrospective cohort study, both R-TKA and C-TKA demonstrated significant and comparable clinical improvements at the one-year primary endpoint. A key finding was that every patient in both surgical cohorts successfully achieved the MCID for the KOOS JR score, indicating an exceptionally high level of postoperative functional recovery regardless of the technique employed. Although the robotic-assisted group exhibited a numerically greater percentage of improvement in joint-specific functional scores, the intergroup analysis revealed that these differences did not reach statistical significance.

These results suggest that while R-TKA provides high surgical precision, both methods are profoundly effective at achieving clinically meaningful outcomes for patients suffering from end-stage osteoarthritis. The comparable rates of MCID achievement across both joint-specific and general health measures (PROMIS PH and PROMIS MH) underscore the efficacy of both approaches in restoring quality of life. Ultimately, while this study demonstrates the short-term success of both techniques, longer-term prospective research is warranted to determine if the high degree of precision offered by robotic technology eventually translates into superior long-term implant survivorship or sustained patient satisfaction.
